# Retinal Distribution and Extracellular Activity of Granzyme B: A Serine Protease That Degrades Retinal Pigment Epithelial Tight Junctions and Extracellular Matrix Proteins

**DOI:** 10.3389/fimmu.2020.00574

**Published:** 2020-04-07

**Authors:** Joanne A. Matsubara, Yuan Tian, Jing Z. Cui, Matthew R. Zeglinski, Sho Hiroyasu, Christopher T. Turner, David J. Granville

**Affiliations:** ^1^Department of Ophthalmology and Visual Sciences, University of British Columbia (UBC), Vancouver, BC, Canada; ^2^International Collaboration on Repair Discoveries (ICORD), University of British Columbia (UBC), Vancouver, BC, Canada; ^3^Department of Pathology and Laboratory Medicine, University of British Columbia (UBC), Vancouver, BC, Canada

**Keywords:** ZO-1, blood-eye barrier, Bruch's membrane, geographic atrophy, soft drusen, choroidal neovascularization, FITC-dextran permeability, ARPE-19

## Abstract

Granzymes are a family of serine proteases first shown to be intracellular initiators of immune-mediated cell death in target pathogenic cells. In addition to its intracellular role, Granzyme B (GzmB) has important extracellular functions in immune regulation and extracellular matrix (ECM) degradation. Verified substrates of extracellular GzmB activity include tight junctional and ECM proteins. Interestingly, little is known about the activity of GzmB in the outer human retina, a tissue in which the degradation of the tight junctional contacts of retinal pigment epithelial (RPE) cells and within the external limiting membrane, as well as remodeling of the ECM in Bruch's membrane, cause the breakdown of the blood-retinal barrier and slowing of metabolite transport between neuroretina and choroidal blood supply. Such pathological changes in outer retina signal early events in the development of age-related macular degeneration (AMD), a multifactorial, chronic inflammatory eye disease. This study is the first to focus on the distribution of GzmB in the outer retina of the healthy and diseased post-mortem human eye. Our results revealed that GzmB is present in RPE and choroidal mast cells. More immunoreactive cells are present in older (>65 years) compared to younger (<55 years) donor eyes, and choroidal immunoreactive cells are more numerous in eyes with choroidal neovascularization (CNV), while RPE immunoreactive cells are more numerous in eyes with soft drusen, an early AMD event. *In vitro* studies demonstrated that RPE-derived tight junctional and ECM proteins are cleaved by exogenous GzmB stimulation. These results suggest that the increased presence of GzmB immunoreactive cells in outer retina of older (healthy) eyes as well as in diseased eyes with CNV (from AMD) and eyes with soft drusen exacerbate ECM remodeling in the Bruch's membrane and degradation of the blood-retinal barrier. Currently there are no treatments that prevent remodeling of the Bruch's membrane and/or the loss of function of the outer blood-retinal barrier, known to promote early AMD changes, such as drusen deposition, RPE dysfunction and pro-inflammation. Specific inhibitors of GzmB, already in preclinical studies for non-ocular diseases, may provide new strategies to stop these early events associated with the development of AMD.

## Introduction

Age-related macular degeneration (AMD) is a complex disease with many risk factors contributing to its pathogenesis (2–4). Despite the fact that clinical and genetic data support an association of a chronic, low-grade inflammatory response in the outer retina during the development of AMD, the exact underlying mechanisms and triggers of inflammation remain elusive (5). Genetic studies point to a central role of the innate immune response and particularly the complement cascade in the development and progression of AMD [for review see (6)]. However, we do not yet understand exactly what causes the earliest signs of inflammation in the retina.

We recently reported that the RPE and immune cells (primarily mast cells) in the choroid express Granzyme B (GzmB) (7), a serine protease that was once thought to function exclusively as intracellular initiators of immune-mediated cell death, capable of inducing apoptosis (a process requiring perforin, a pore-forming protein) in target cells. Earlier studies in non-ocular systems identified that GzmB is located on chromosome 14 in both human (14q.11.2) and mouse genomes. Several cytotoxic [natural killer (NK), CD8+, T], non-cytotoxic immune (mast, macrophages, basophils), and non-immune cells (spermatozoa, keratinocytes) express GzmB. In addition to its well-established intracellular role, GzmB also has important extracellular functions in immune regulation and the degradation of extracellular matrix (ECM) proteins (8–12). Experimental degranulation of choroidal mast cells in rodents resulted in RPE abnormalities and outer retinal barrier breakdown (13), however it is not yet known which of the many proteases, or other choroidal mast cell mediators, are involved (14).

**Figure 1:**
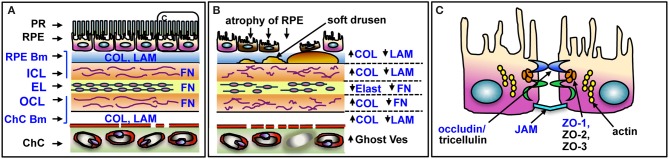
Schematic of outer retina and substrates of GzmB in BM and RPE. **(A)** The five layers of BM and major ECM proteins are shown in blue text. The PR and RPE sit above the BM on the RPE basement membrane. The choriocapillaris sits below BM, on its basement membrane. **(B)** Changes in outer retina associated with AMD pathology. RPE undergo atrophy and cell death, soft drusen deposits accumulate below RPE. Laminin, elastin, and fibronectin undergo cleavage resulting in overall ECM fragility; altered forms of collagen increase, causing thickening of BM. Choriocapillaris undergoes atrophy, with closure of some vessels, identified as ghost vessels. **(C)** Enlargement of box in **(A)** depicting tight junctional and cell adhesion proteins on RPE cleaved by GzmB in blue text. PR, photoreceptors; RPE, retinal pigment epithelium; RPE Bm, basement membrane of RPE; ICL, inner collagenous layer; EL, elastin layer; OCL, outer collagenous layer; ChC Bm, basement membrane of the choriocapillaris; COL, collagen; LAM, laminin; FN, fibronectin; Ghost Ves, closed lumen of vessel with dead endothelial cells; JAM, junctional adhesion molecules; ZO, zonula occludins. Adapted from Nita et al. (1).

Here we report the age-related changes and cellular distribution of GzmB in the healthy and diseased human outer retina, and *in vitro* evidence for GzmB's extracellular role in the disruption of the outer blood-retinal barrier (oBRB) function by cleavage of tight junctional proteins between retinal pigment epithelial (RPE) cells and ECM proteins in Bruch's Membrane (BM). BM is an important outer retinal ECM that regulates the exchange between the (1) metabolically active combination of photoreceptor and RPE and (2) the choriocapillaris blood supply. Several of the ECM proteins within BM are known substrates for extracellular GzmB activity, including fibronectin (FN), vitronectin (VN), and laminin (LAM) and a small subset of collagens (COL) (1, 15–18) (Figure 1). The remodeling of BM during aging and AMD is known to also affect RPE cell adhesion and function, which in turn, compromises oBRB function (18, 19). In addition to the breakdown of BM, outer retina is also compromised by the loss of function of the oBRB, which is maintained by the tight junctional contacts between RPE cells. Given that the breakdown of BM and loss of oBRB function are associated with the earliest events in the development of AMD (1, 19–21), we speculate that GzmB activity may promote early changes in outer retina that contribute to AMD development.

Our earlier work showed that GzmB cleaves ECM in non-ocular systems, implicating extracellular GzmB activity in pathological chronic inflammation, delayed wound healing, skin injuries, and cardiopulmonary disease (8–10, 12, 22). Here we address the evidence that age-related increases in extracellular GzmB in the outer retina promote pathological remodeling of the BM and alterations in RPE barrier function. Given that there are no treatment strategies that prevent pathological breakdown of BM or the oBRB, studies on the extracellular GzmB activity in ocular tissues may allow us further insights into novel immune-mediated mechanisms associated with degradation and remodeling of the outer retina during aging and AMD development.

## Materials and Methods

Sixteen pairs of non-diseased donor eyes, consented for research, were obtained from the Eye Bank of British Columbia (EBBC, Vancouver, British Columbia, Canada). Healthy eyes collected within 12 h following death were immersed in 10% buffered formalin after corneal tissues removed for corneal transplant purposes. Each normal donor eye had a medical/hospital history obtained by EBBC, and these eyes, to the best of our knowledge, were ocular disease free. These eyes were then embedded in paraffin and serial sections at 6 micra thickness were obtained. Every 20th section was stained for H & E and assessed by light microscopy. Tissue sections from the macular area were assessed to identify and screen for ocular pathologies. Healthy eyes had normal retinal organization, and did not display outer retinal fibrosis, RPE/PR degeneration, neovascularization, or hemorrhage. Healthy donor tissues with the following pathologies were excluded in this study: local or systemic infection, progressive brain pathologies, systemic diseases of unknown origin, lymphoproliferative, or myeloproliferative disorders or any intrinsic eye disease.

Diseased eyes with geographic atrophy (GA), choroidal neovascularisation (CNV), or soft drusen deposits were obtained from the Department of Pathology, UBC. Tissue sections from the macular area were stained by hematoxylin and eosin, and then assessed by ophthalmic pathologists to identify and screen for ocular pathologies. In total, 8 eyes with GA (from AMD), 6 eyes with CNV (from AMD), 9 eyes with numerous soft drusen (from non-AMD eyes) were included in this study. For cell culture studies, ARPE-19 (RPE cell line, ATCC) and primary fetal RPE cells were used. Primary RPE were isolated from human fetal donor eyes, with no known pathology, that were consented for research studies from the CARE program at BC Women's Hospital under Clinical Research Ethics Board (UBC) approval.

### Immunohistochemistry

#### Human Outer Retina

The immunohistochemical procedures and analysis followed those previously described (23). Briefly, deparaffinized tissue sections (6 μm thickness) were probed with a primary antibody against GzmB (Abcam), followed by incubation in secondary antibody and developed in ABC–AEC system (Vector Labs). All sections were taken from central macular area of the retina and each cross section used in this analysis contained the optic nerve head to verify its retinal location. A list of primary and secondary antibodies, source, and dilutions is shown in Table 1. The immunoreactive RPE and immune cells in the choroidal layers were counted and normalized to 1,050 μm length units of BM. Approximately 4–6 sections per donor eye were analyzed. The immunoreactivity was compared among eyes from healthy older, healthy younger, GA, CNV, or soft drusen (an early marker of AMD) donors.

**Table 1:** List of antibodies used.

**Antigen**	**Antibody (Catalog No.)**	**Dilution**	**Source**
GzmB	Rabbit polyclonal (ab4059)	1:100	Abcam, Burlingame, CA
ZO-1	Rabbit polyclonal (61-7300)	1:100	Thermo Fisher Scientific, Waltham, MA
JAM-A	Mouse monoclonal (14-3219-82)	1:100	Thermo Fisher Scientific, Waltham, MA
Occludin	Mouse monoclonal (E-5. SC-133256)	1:100	Santa Cruz Biotechnology, Dallas TX
Vinculin	Rabbit monoclonal (ab129002)	1:1,000	Abcam, Burlingame, CA
Fibronectin	Rabbit polyclonal (ab2413)	1:1,000	Abcam, Burlingame, CA
Laminin 5	Rabbit polyclonal (ab14509)	1:1,000	Abcam, Burlingame, CA
Collagen IV	Mouse monoclonal (G-2. SC-398655)	1:1,000	Santa Cruz Biotechnology, Dallas TX
Collagen I	Mouse monoclonal (E-6. SC-393573)	1:1,000	Santa Cruz Biotechnology, Dallas TX
CD68	Mouse monoclonal (M0876)	1:100	Dako Carpinteria, CA
Tryptase	Mouse monoclonal (ab2378)	1:100	Abcam, Burlingame, CA
Rabbit IgG Horseradish Peroxidase conjugated Antibody	Polyclonal Goat IgG (HAF008)	1:1,000	R&D Systems, Minneapolis, MN
Mouse IgG Horseradish Peroxidase conjugated Antibody	Polyclonal Donkey IgG (HAF018)	1:1,000	R&D Systems, Minneapolis, MN

#### ARPE-19 or Primary RPE Cells Grown in Chamber Slides or Transwell Inserts

Tight junctional proteins were visualized by immunofluorescence on both ARPE19 or primary RPE cells grown to confluence and stimulated in chamber slides. Tight junctional proteins were evident on ARPE19 (within 5–7 days) and on primary RPE (within 7–10 days) in culture. After GzmB stimulation, cells were fixed *in situ* with cold methanol, washed with PBS, and incubated in 2% normal goat serum (Vector Labs) for 30 min at room temperature (RT). Next, chamber slides were incubated with antibody against ZO-1 (Invitrogen), JAM-A (Invitrogen), or Occludin (Santa Cruz). All antibodies were diluted at 1:100 in PBS with 0.1% TX-100 and chamber slides were incubated for 1 h at RT, then overnight at 4°C. Primary antibodies were omitted for negative controls. Next, slides were thoroughly washed with PBS, and then incubated with Alexa 488 conjugated goat anti-rabbit or anti-mouse secondary antibody (1:400) for 1 h at RT. Slides were washed with PBS, and then incubated with Hoechst 33258 (DAPI, Sigma, 1:500) for 20 min. A blade was used to remove chambers and silicon tapes, and marker pen was used to label the locations of different chambers on slides. Slides were mounted with glycerol (50:50 glycerol: PBS) and 1.5 mm coverslips. For transwell inserts, after GzmB stimulation and measurements of FITC-dextrans fluorescence, membranes at the bottom of inserts were cut out and adherent ARPE-19 cells underwent immunocytochemistry with ZO-1 primary antibody as above. An LSM 510 confocal laser-scanning microscope was used to image antibody labeling at 488 and 543 nm, DAPI (nuclei) was imaged at 405 nm and images stored as digital files. All settings on confocal were kept constant throughout the imaging sessions in order to compare intensity of fluorescent signals between groups.

### Cell Culture

Primary RPE were grown as described previously (24). Briefly, passage 5–7 ARPE-19 cells (or passage 3–5 primary RPE cells) were seeded into 6 well plates in 1.0 mL Dulbecco's modified Eagle Medium/F12 medium (DMEM, Life Technologies) containing 10% fetal bovine serum (FBS), 100 U/mL penicillin and 100 μg/mL streptomycin and kept in a humidified chamber with 5% CO_2_ at 37°C until confluent. Cells were washed with PBS and starved for 3 h in serum free DMEM before GzmB stimulation. GzmB was isolated YT cells, an autonomously proliferating human NK cell line devoid of GzmA and GzmK activity, and procedures were modified from Shi et al. (25). Next, DMEM medium with 0 nM (control) or 100 nM GzmB was added to wells and cells cultured at 37°C incubator for 5 h. After 5 h, supernatants were carefully removed and stored, and cells rinsed with PBS on ice. Total protein lysates were collected for western blot analysis. To determine effects of exogenous GzmB on the cleavage of RPE-derived ECM proteins WBs were undertaken using both supernatant and lysate samples.

In some studies, ARPE19, a human RPE cell line, were used at passage 5–7 (26). These cells were grown to confluence on chamber slides or transwell inserts. Briefly, ~1.6 × 10^5^/cm^2^ cells were seeded in a laminin-coated Transwell insert (0.4 μm pore size, 12 mm diameter, Fisher Scientific) or on chamber slides (LabTek II) in 0.2 mL DMEM containing 10% FBS, 100 U/mL penicillin and 100 μg/mL streptomycin and kept in a humidified chamber with 5% CO_2_ at 37°C. Next, the cells were washed with PBS, starved for 3 h in serum-free DMEM before GzmB stimulation. DMEM medium with GzmB (0, 1, 10, 50, 100) was added to culture slides for 5 h at 37°C. After 5 h, culture medium was removed and stored; cells were fixed *in situ* with 100% methanol on ice for 15 min.

#### MTT Cell Viability Assay

ARPE-19 cells were grown to 95% confluence in 96-well plates for MTT assays. Cells were then washed with PBS twice and then starved for 3 h in serum-free DMEM before GzmB stimulation at different concentrations (0, 10, 20, 50, 100 nM). Next, the 96-well plates were placed in 37°C incubator for 24 h. After 24 h, culture medium was removed, and 250 μl of 0.5 mg/ml MTT buffer (1:10 dilution in DMEM) was added to each well, incubated at 37°C for 2 h. MTT solution was removed, 250 μl DMSO added to each well and reincubated at 37°C for 15–20 min; next, absorbance at 570 nm wavelength was read using the Hybrid Multi-Mode Reader (BioTek Synergy H1).

#### Western Blot

To detect the cleaved products in primary RPE cultures after GzmB stimulation, confluent primary RPE cells were first starved (as above), then treated with GzmB (0 or 100 nM) in 37°C incubator for 5 h. After 5 h, culture supernatants were collected and aliquoted for later analysis, and RPE cells rinsed with PBS twice on ice. Next, the adherent cells were treated with 200 μl of lysis buffer [10 ml RIPA lysis buffer with proteinase inhibitor cocktail (Roche)] and cell lysates collected from each well, centrifuged for 10 min at 4°C, and aliquoted. Cell lysates were quantified with a BCA Assay (Pierce, Thermo Fisher) for total protein concentrations and run on gels under reducing conditions. Established blotting procedures were followed to visualize the proteins of interest. A list of primary and secondary antibodies, source and dilutions used in western blot is shown in Table 1. Targeted proteins on membranes were detected with Pierce™ ECL Western Blotting Substrate Kit (Thermo Fisher). Protein band intensity was measured using Image J (NIH). Vinculin, a high molecular weight (MW = 124 kDa) housekeeping gene, was used for quality control on supernatant samples.

#### FITC-Dextran Permeability Assay

Primary RPE cells were grown to confluence in transwell system inserts for 24-well plates for a functional assay of solute flux through the monolayer. For dextran permeability assay, the transwells were washed with PBS, and starved for 3 h in serum-free DMEM before GzmB stimulation. DMEM with 0 nM (controls) or 100 nM GzmB was placed in the upper insert of the transwell systems at 37°C for 5 h. After 5 h, the culture medium was removed without disturbing cells, and DMEM culture medium with 1 mg/ml FITC-dextran (70 kDa, Sigma) solution was added into all upper inserts for another 5 h. After 5 h, all inserts were removed and fluorescence intensity at 490 nm (excitation)/520 nm (emission) wavelengths was read using the Hybrid Multi-Mode Reader (BioTek Synergy H1). For trans-epithelial resistance measurements, transwells were placed in electric cell substrate impedance sensing apparatus, media changed to serum free and allowed to stabilize before addition of 0 nM (controls) or 50–100 nM GzmB in to lower or upper compartment. Averaged measurements of impedance, capacitance are graphed for 0–24 h.

### Statistical Analysis

Data are presented as mean ± SEM. All experiments were repeated in triplicate or quadruplicate. Statistical analysis was performed using Prism Ver8 (GraphPad Software). To compare two groups, independent sample *T*-test was used for western blot and immunohistochemistry analysis of human tissue samples. To compare more than two groups, a one-way ANOVA test with Tukey's multiple comparisons *post-hoc* test was used. Statistical significance level was set at *p* < 0.05.

## Results

### GzmB Immunohistochemistry in Human Outer Retina

**Figure 2:**
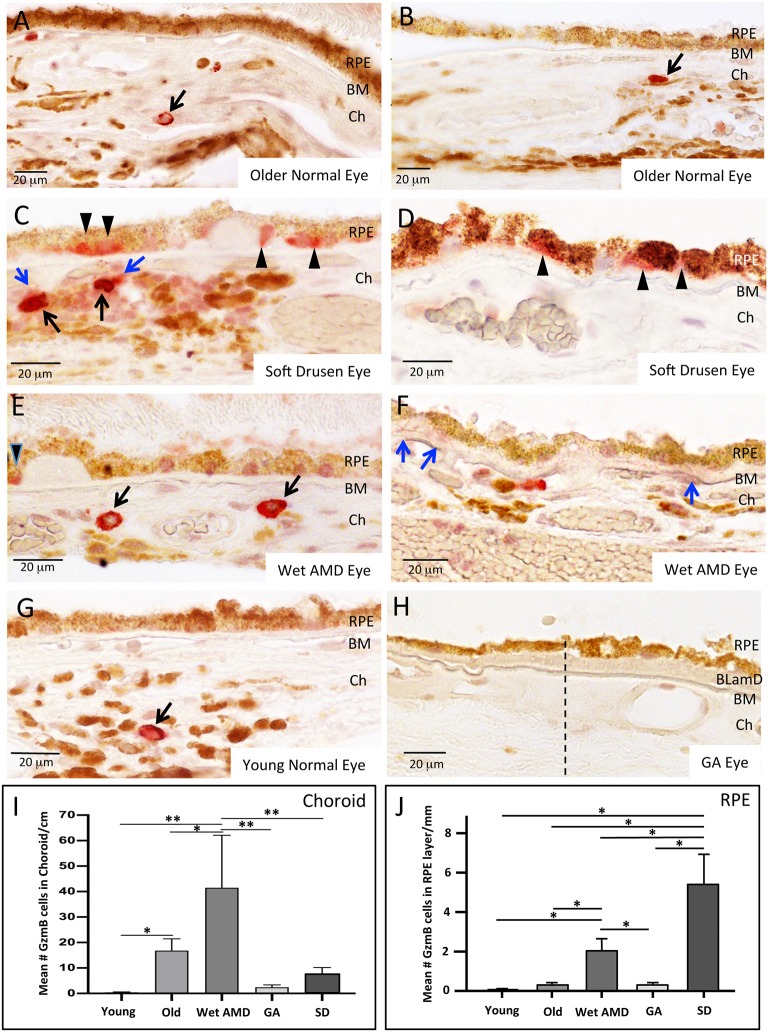
GzmB Immunoreactivity in normal and diseased human outer retina. **(A,B)** Examples of GzmB immunoreactivity in an older normal eye. Strong labeling with amino-ethyl carbazole chromogen (red) indicates GzmB+ cells in the choroid (black arrows). RPE cells in older normal eyes rarely displayed GzmB immunoreactivity. **(C,D)** Examples of GzmB immunoreactivity in eyes with soft drusen, an early marker of AMD. Strong labeling with amino-ethyl carbazole chromogen (red) indicates GzmB+ cells in the choroid (black arrows), with a cloud of extracellular GzmB immunolabeling surrounding the choroidal mast cells (blue arrows). The basal compartment of the RPE cell near soft drusen sites also contains GzmB immunoreactivity (black arrowheads). Note GzmB+ RPE appear of the “non-uniform” phenotype (27). **(E,F)** Examples of GzmB immunoreactivity in a wet AMD eye. Strong labeling with amino-ethyl carbazole chromogen (red) indicates GzmB+ cells in the choroid (black arrows). Extracellular GzmB immunolabeling is evident in the basal laminar deposit (blue arrows) of a wet AMD eye. In wet AMD eyes, fewer RPE cells contained GzmB compared to eyes with soft drusen, however, one is shown in **(E)** near a drusen site (black arrowhead). **(G)** An example of GzmB immunoreactivity in a younger normal eye demonstrating a single choroidal mast cell (arrow). RPE cells in younger normal eyes rarely displayed GzmB immunoreactivity. **(H)** GzmB immunoreactivity in a GA eye. Note lack of red chromogen product in choroid and in RPE. Dash line indicates a change in the morphology of the RPE monolayer, with “non-uniform” and “dissociated” RPE phenotypes to the right of the dashed line (27). Note thickened basal laminar deposit (BLamD) and deterioration of the choriocapillaris. Additional images of a GA eye are shown in Supplementary Figure 6. **(I)** Analysis of GzmB immunoreactivity of choroidal cells in older (>65 years, *N* = 8) and younger (<55 years *N* = 8) normal donor eyes, wet AMD (*N* = 6), GA (*N* = 8), and soft drusen eyes (*N* = 9). The number of GzmB+ choroidal cells was significantly higher in wet AMD eyes compared to all other groups, while the number of GzmB+ choroidal cells in older eyes was significantly higher than younger eyes. One-way ANOVA Test and Tukey's multiple comparisons *post-hoc* test (*p < 0.05; **p < 0.01). **(J)** Analysis of GzmB immunoreactivity of RPE cells in older (>65 years, *N* = 8) and younger (<55 years *N* = 8) normal donor eyes, wet AMD (*N* = 6), GA (*N* = 8), and soft drusen (*N*=*9*). The number of GzmB+ RPE cells was significantly higher in soft drusen compared to all other groups, while the number of GzmB+ RPE cells in wet AMD eyes was significantly higher than younger, older and GA eyes, but significantly lower than soft drusen eyes. One-way ANOVA Test and Tukey's multiple comparisons *post-hoc* test. (*p < 0.05). Scale bar: 20 μm.

Our earlier work showed that GzmB accumulates in cardiovascular, pulmonary and skin tissues with aging and/or chronic inflammation (10, 11, 28). Here, we assessed GzmB immunoreactivity in outer retina of postmortem eyes from older (>65 years) and younger (<55 years) donors. We assessed the GzmB+ cells in the choroidal stroma only and omitted blood cells that were clearly in vasculature. The older donor eyes had more GzmB+ labeling than younger eyes, consistent with our earlier findings in the retina of aged mouse models (7), (Figures 2A,B,G,I). The majority of GzmB+ cells were choroidal mast cells (identified by toluidine blue staining, Supplementary Figure 1) in the choriocapillaris and Sattler's layer, however within the choroidal stromal layers, a small number of macrophages (identified by CD68 immunoreactivity, Supplementary Figure 4) were also GzmB+. RPE were immunoreactive for GzmB, with slightly increased numbers in older donors. However, unlike the choroidal mast cells, the number of GzmB immunoreactive RPE in younger and older healthy donors did not reach significance (Figures 2I,J).

Next, we assessed the distribution of GzmB+ choroidal cells in donor eyes with geographic atrophy (GA from AMD), eyes with the exudative (wet) form of AMD and eyes with soft drusen (SD, an early precursor to AMD pathology). Wet AMD eyes contained greater numbers of GzmB+ choroidal cells compared to GA (Figures 2E,H, Supplementary Figure 6) or soft drusen eyes (Figures 2C,D,I). Extracellular GzmB labeling was also evident in the BM and basal laminar deposits in wet AMD eyes and in GA eyes (Figure 2F, Supplementary Figures 5, 6). In the CNV lesion of a wet AMD eye, extracellular GzmB immunoreactivity was present in the CNV lesion near hypertrophied RPE (Supplementary Figure 5).

The pattern of GzmB labeling in RPE cells amongst the donor eye groups was different from that observed for GzmB+ choroidal cells (Figure 2J). Eyes with soft drusen had higher numbers of RPE cells expressing GzmB than the CNV or GA eyes (Figures 2C–F,H). The RPE cells expressing GzmB were often spatially located near soft drusen deposits (Figures 2C–E). However, GzmB immunoreactivity was not located in RPE near hard drusen deposits (Supplementary Figure 5). Those RPE cells, which were immunoreactive for GzmB, usually had labeling in the basal cytoplasmic compartment of the RPE (Figures 2C–E) and appeared to belong to the categories of “non-uniform” RPE phenotypes (27).

**Figure 3:**
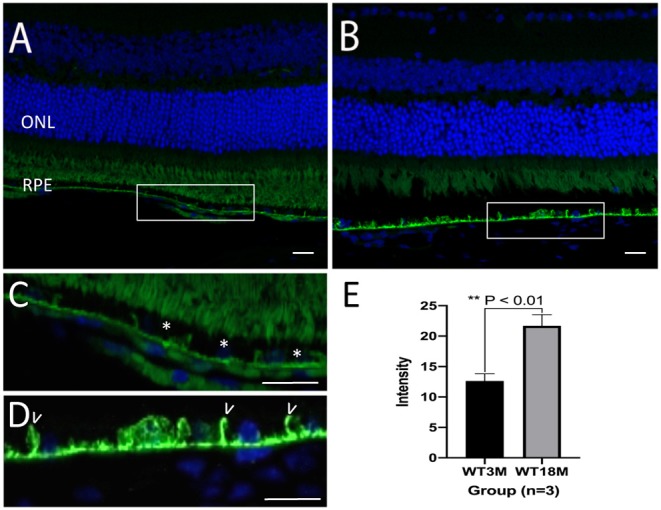
Immunreactivity of GzmB in Bruch's Membrane and RPE layer of a young (3 month) and old (18 month) C57Bl/6J mouse retina. **(A)** GzmB immunoreactivity (green, 488 nm) is lower in mouse retina from 3 month old mouse compared to **(B)** an 18 month old mouse. Boxed areas are shown at higher power in **(C)** with GzmB punctate labeling (*) on Bruch's membrane of 3 month old, and in **(D)** which demonstrates stronger immunofluorescence intensity along BM and between RPE cell borders (some shown by white arrowheads) of the older mouse retina. **(E)** Independent-sample *T*-test (*n* = 3) between two groups shows the increased GzmB level in BM and RPE layers of 18M mice: mean ± SEM; *p < 0.01. DAPI (405 nm) labeling of nuclei is shown in blue. Scale bar = 20 μm.

In C57Bl/6J mice, GzmB immunoreactivity was present in outer retina, specifically in the extracellular matrix of BM and the intercellular spaces between RPE cells (Figures 3A–D). In the mouse models, there was an age-dependent increase in GzmB immunoreactivity (Figure 3E).

### Extracellular GzmB Cleaves RPE Tight Junctional and Cell Adhesion Proteins

**Figure 4:**
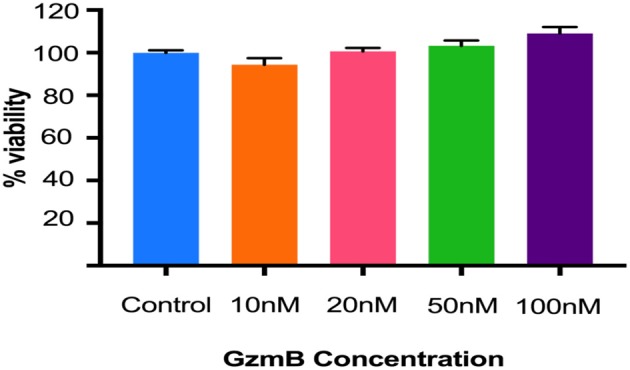
GzmB does not affect the viability of ARPE-19 cells. Cells were treated with exogenous GzmB (10, 20, 50, 100 nM) for 24 h and compared to controls (GzmB = 0 nM). No significant changes were observed in viability at any of the GzmB concentrations. Data were analyzed using one-way ANOVA test and Tukey's multiple comparisons *post-hoc* test (mean ± SEM, *N* = 5 per group).

**Figure 5:**
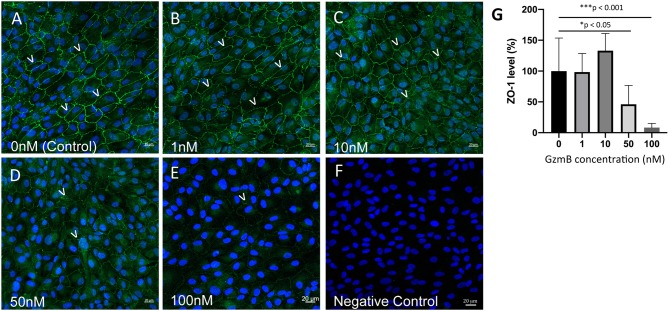
Reduced immunoreactivity of ZO-1 in ARPE-19 cells stimulated by exogenous GzmB. **(A–E)** Note immunoreactivity of ZO-1 tight junctional contacts (green, 488 nm) between cells (some shown by white arrowheads) is reduced with increasing concentrations of GzmB (1, 10, 50, 100 nM) for 6 h. **(F)** Omission of primary antibody demonstrates lack of green ZO-1 immunolabeling. **(G)** Significant differences were observed between controls and 50 and 100 nM GzmB. A one-way ANOVA and Tukey's multiple comparisons *post-hoc* test (*N* = 6 per group) were used to compare control group with GzmB-treated groups: mean ± SEM; *p < 0.05; ***p < 0.001. DAPI (405 nm) labeling of nuclei is shown in blue. Scale Bar = 20 μm.

**Figure 6:**
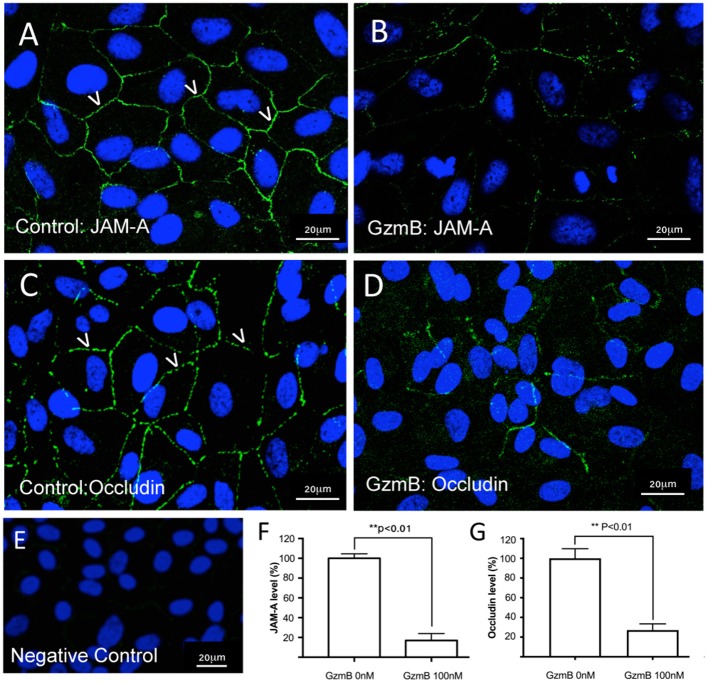
Reduced immunoreactivity of JAM-A and Occludin in ARPE-19 cells stimulated by exogenous GzmB. **(A)** JAM-A immunoreactivity demonstrates tight junctional contacts (green 488 nm, some shown by white arrowheads) on RPE in controls wells (GzmB = 0 nM). **(B)** After 6 h with GzmB stimulation (100 nM), JAM-A immunoreactivity is reduced on ARPE-19. **(C)** Occludin immunoreactivity between cells (some shown by white arrowheads) in control wells (GzmB = 0 nM). **(D)** After 6 h with GzmB stimulation (100 nM), Occludin immunoreactivity is reduced on ARPE-19. **(E)** Omission of primary antibody demonstrates no immunoreactivity. Significant differences were observed for **(F)** JAM–A and **(G)** Occludin using an independent-sample *T*-Test (*N* = 6 per group) between control and GzmB-treated group: mean ± SEM; **p < 0.01. Images were taken from ARPE-19 cells and are representative of primary RPE experiments undertaken at the same time. DAPI (405 nm) labeling of nuclei is shown in blue. Scale bar = 20 μm.

*In vitro* studies were undertaken to assess the impact of exogenous GzmB on RPE cell cultures. First we assessed the effect of GzmB on cell viability using an MTT assay. ARPE-19 cells stimulated with exogenous GzmB for 24 h, at concentrations from 10 to 100 nM, demonstrated no changes in cell viability compared to controls (GzmB at 0 nM) (Figure 4). Next, ARPE-19 cells were grown and stimulated with GzmB at 1, 10, 50, and 100 nM for 6 h in chamber slides to assess the degradation in tight junctional protein ZO-1. Significant degradation of ZO-1 was observed at 50 and 100 nM GzmB as shown by loss of immunofluorescence (Figures 5A–G). Exogenous GzmB stimulation at 100 nM also cleaved and degraded JAM-A and Occludin, two additional tight junctional proteins on ARPE-19, as shown by loss of immunofluorescence (Figures 6A–G). These data extend earlier work by demonstrating that tight junctional proteins from an ocular source (e.g., RPE-derived) are substrates of GzmB.

### Extracellular GzmB Causes Increased Dextran Flux in Confluent ARPE-19 Cultures

**Figure 7:**
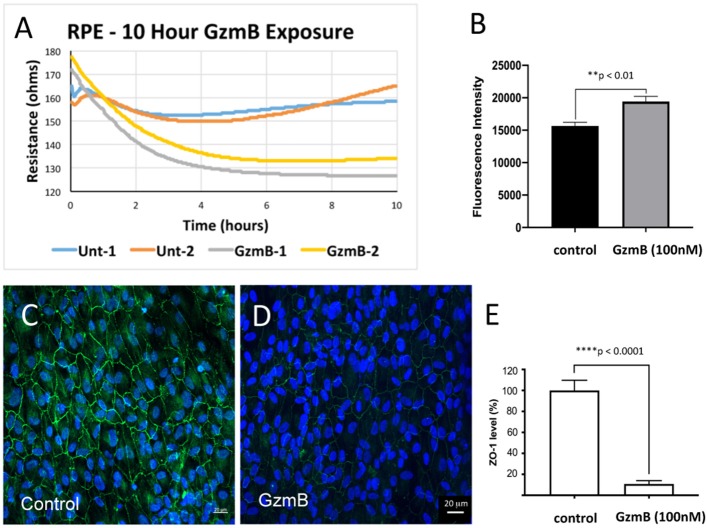
Lowered trans-epithelial resistance and increased FITC-dextran flux after GzmB stimulation on primary RPE cells grown on transwell inserts. To test the primary RPE barrier function, cells were grown on transwell inserts and stimulated with GzmB at 100 nM. **(A)** Trans-epithelial resistance measurements on resting cells ranges from 160 to 180 ohms. After initiation of GzmB stimulation (time = 0), the resistance of RPE cultures decreases to 135–125 ohms (time = 10 h) as shown by yellow and gray traces. The control cells maintained resistance at 160–165 ohms (time = 10 h) as shown by the blue and orange traces. **(B)** FITC-dextran flux assay demonstrated increased FITC-dextran fluorescence intensity measured in the lower compartment of GzmB stimulated wells compared to control wells. **(C–E)** After the flux assay, the membranes on which cells were attached were cut out and subjected to immunocytochemistry. Strong ZO-1 immunolabeling on primary RPE cells on transwell insert membrane in control wells (0 nM GzmB) after 5 h shown in **(C,D)** in experimental wells (100 nM GzmB) after 5 h of GzmB stimulation. **(E)** The ZO-1 immunoreactivity was quantified in controls and GzmB stimulated wells and an independent-sample *T*-Test (*N* = 6) between control and GzmB-treated group was undertaken and graphed as mean ± SEM; ****p < 0.0001; **p < 0.01. DAPI (405 nm) labeling of nuclei is shown in blue.

It is hypothesized that the functional significance of GzmB cleavage of cell junctional proteins on RPE leads to the disruption of the blood-eye barrier, an early event in the pathogenesis of AMD (1, 17, 29–31). We tested the barrier function of primary RPE cells grown on transwell inserts by measuring fluorescence associated with movement of FITC-labeled dextrans (70 kDa) across the RPE monolayer, an established method and indicator of solute flux used routinely to test barrier function of RPE (32–34). Fluorescent dextrans were placed in the upper compartment and measured in the lower compartments after exogenous GzmB (100 nM) stimulation for 5 h (Figures 7B–E). After 5 h, fluorescent intensity was higher in lower wells of primary RPE cells stimulated with GzmB compared to controls (*p* < 0.01) (Figure 7B). Subsequent ZO-1 immunohistochemistry on primary RPE cells attached to the membrane of the transwell insert after GzmB stimulation demonstrated a loss of ZO-1 immunofluorescence intensity (Figures 7C–E), consistent with an increased permeability, as indicated by the observed increase in the FITC-dextran fluorescence in the lower compartment. We also tested the trans-epithelial resistance of the RPE monolayer grown on transwells for 10 h after exogenous GzmB (100 nM) stimulation (Figure 7A). Note that the resistance is lowered by exogenous GzmB (gray and yellow traces), while control (unstimulated) RPE retained their resistance measurements at ~160 ohms (blue and orange traces).

### Extracellular GzmB Degrades Primary RPE-Derived ECM Proteins

**Figure 8:**
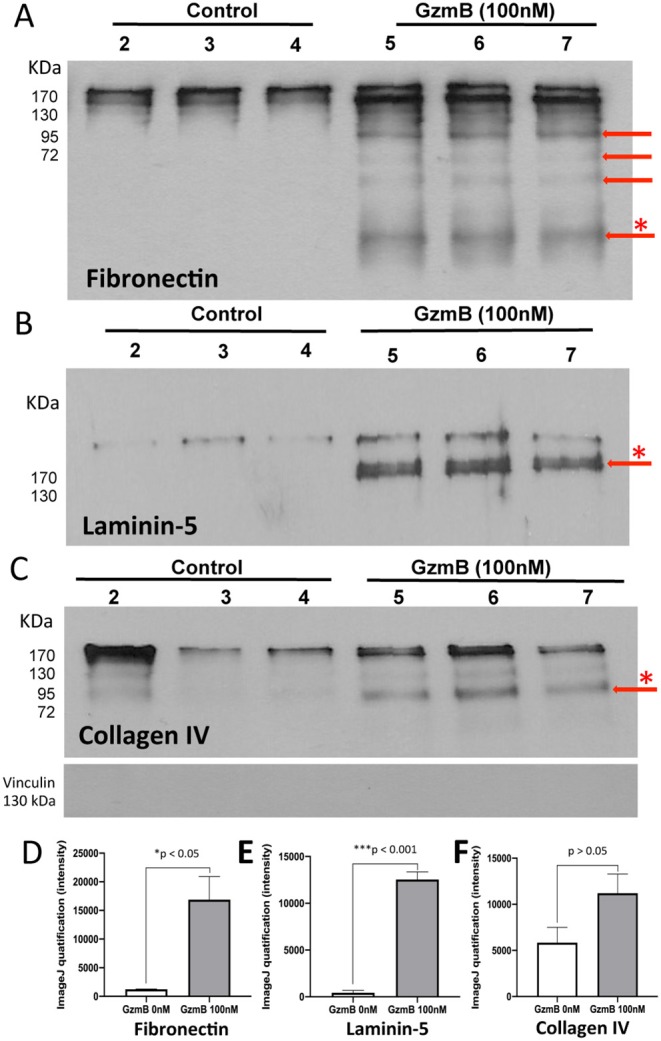
Western blots reveal cleavage of primary RPE-derived extracellular matrix proteins by exogenous GzmB. Primary RPE cells were stimulated with GzmB (100 nM, 5 h), and both protein lysates and culture supernatant were collected. Western blot of supernatant samples were processed. **(A)** Western blot using antibody against fibronectin shows cleavage bands due to GzmB stimulation of primary RPE with 0 nM GzmB (controls, lanes 2–4) or 100 nM exogenous GzmB (lanes 5–7). Note cleavage bands at lower molecular weight, some identified by red arrows. The densiometric analysis of the band with asterisk (*) is graphed in **(D)** and demonstrates significant differences between control and 100 nM GzmB groups. **(B)** Western blot using antibody against Laminin-5 (now known as Laminin-332) shows supernatants from cultures of primary RPE with 0 nM (controls, lanes 2–4) or 100 nM exogenous GzmB (lanes 5–7). Note cleavage band, identified by red arrow, in samples from GzmB stimulation group (lanes 5–7), but not control lanes (2–4). The densiometric analysis of the band with asterisk (*) is graphed in **(E)** and demonstrates significant differences between control and 100 nM GzmB groups. **(C)** Western blot using antibody against Collagen IV shows supernatants from cultures of primary RPE with 0 nM (controls, lanes 2–4) or 100 nM exogenous GzmB (lanes 5–7). Note cleavage band, identified by red arrow, in samples from both GzmB stimulation group and controls. The densiometric analysis of the band with asterisk (*) is graphed in **(F)** and demonstrates no significance between control and 100 nM GzmB groups. Vinculin (124 kDa, housekeeping gene) antibody was used for quality control and demonstrates supernatant samples did not contain cell lysates.

Our earlier studies reported that GzmB cleaves ECM in non-ocular systems, implicating extracellular GzmB activity in pathological chronic inflammation, delayed wound healing, skin injuries, and cardiopulmonary disease (8–10, 12, 22). Age-related increases in GzmB (Figure 2), may promote the remodeling of Bruch's Membrane, an important ECM in outer retina. We studied the effect of exogenous GzmB on RPE-derived FN, LAM-5 (now known as LAM-332), COL-I and COL-IV. Primary RPE cultures were grown to confluence and stimulated with exogenous GzmB (0 nM controls vs. 100 nM GzmB) for 5 h. Supernatant samples containing secreted ECM proteins were probed with primary antibodies against FN, LAM-5 and COL-IV by western blot, and revealed several cleaved bands in those samples stimulated with 100 nM GzmB compared to controls (0 nM GzmB) (Figures 8A–C). Densitometric analysis revealed that the cleaved bands for FN and LAM-5 reached significance (*p* < 0.05), while that of COL-IV did not (*p* > 0.05) (Figures 8D–F). There were no cleaved bands observed for COL-I (Supplementary Figure 2), consistent with the earlier finding that COL-1 is not a substrate of GzmB (15, 16).

## Discussion

GzmB contributes to the pathology of autoimmune and/or chronic inflammatory conditions through the degradation of extracellular proteins and tissue matrices (8–10, 12, 22). Here we report GzmB expression in the healthy and diseased human retina and *in vitro* studies demonstrating the effects of exogenous GzmB on RPE-derived tight junctional and ECM proteins. Our premise is that extracellular GzmB contributes to the onset and/or progression of AMD via the cleavage of key extracellular proteins in BM resulting in RPE dysregulation and barrier function abnormalities. Later stages of AMD including RPE atrophy and remodeling of BM with the release of sequestered angiogenic and pro-inflammatory factors, may develop gradually, in part due to the unchecked activity of extracellular GzmB.

### GzmB Immunoreactivity in the Outer Retina

Aging is the major risk factor for AMD. The molecular inflammation (e.g., inflammaging, para-inflammation) hypothesis of aging suggests that by-products of low-grade inflammatory processes may accumulate in tissues, and serve as a bridge between normal aging and age-related pathological processes (35, 36). The human outer retina demonstrated a significant age-related increase in the number of GzmB+ cells, specifically in the choroid. This was consistent with our preliminary findings of an age-related increase in GzmB immunoreactivity in mouse outer retina, specifically the BM in mouse models (7) (Figure 3). In the mouse retina, GzmB immunoreactivity is evident in the extracellular matrix of BM and the intercellular spaces between RPE cells. This is in contrast to the findings in human tissues presented here, where the majority of immunoreactivity was intracellular, However, sparse extracellular GzmB immunoreactivity was evident in wet AMD eyes, in a CNV lesion in a wet AMD eye, and in the choroids of a soft drusen eye and a GA eye (Figure 2C, Supplementary Figure 5). Our human data allowed us to identify the cells that produce GzmB. Choroidal mast cells appear to be the major source of GzmB especially in CNV eyes. RPE cells appear to be an important source of GzmB in eyes with soft drusen and CNV. We hypothesize that GzmB's extracellular role in cleavage of the ECM causes the breakdown of the oBRB, an early event in the development of AMD. This is supported by our *in vitro* studies in which we demonstrated the cleavage of tight junctional and cell adhesion molecules as well as RPE-derived ECM proteins.

Interestingly, earlier reports of tryptase, another serine protease produced by mast cells in the choroid, has also been implicated in aging and the atrophic form of AMD leading to the GA in its late stage (29, 37). While tryptase is associated with GA, our work demonstrated that GzmB is rarely present in GA eyes, but more significantly in CNV and soft drusen eyes (Figures 2G,H, Supplementary Figure 6). Preliminary results suggest that different populations of mast cells are responsible for their secretion (Supplementary Figure 3). It is of note that GzmB is present in RPE cells (Figure 2), while tryptase is not produced by RPE, and importantly, tryptase does not cleave tight junctional proteins ZO-1 or JAM-A (38). Future studies will focus on the dual effects of these mast cell mediators toward understanding their separate or combined role in the development of AMD.

Next, we undertook *in vitro* cell studies to assess the role of exogenous GzmB on RPE-derived proteins, as immunoreactivity patterns can provide spatial localization within the outer retina, but does not allow for functional analysis. Of note, concentrations of exogenous GzmB, up to 100 nM, did not affect RPE cell viability. As such, we focused on the effects of GzmB cleavage on RPE cell-derived proteins.

### GzmB Cleaves RPE Tight Junction and Cell Adhesion Proteins

RPE-derived tight junctional and cell adhesion proteins, ZO-1, JAM-A, and Occludin, were degraded by the application of exogenous GzmB *in vitro*. These tight junctional and adhesion proteins are essential components of the oBRB and ocular substrates GzmB. Thus, it is likely that during the aging process, tight junctional proteins may be subject to slow degradation due to increased age-related accumulation of extracellular GzmB (30, 31, 39). Earlier *in vivo* studies using lentiviral vector knockdown of ZO-1 in C57BL mice showed that loss of ZO-1 on RPE caused abnormal retinal barrier function, RPE proliferation and clumping, pyknosis, and eventual RPE death (30). In another study, ZO-1 and JAM-A were shown to deteriorate on RPE in a light-induced mouse model of AMD, which resulted in RPE atrophy (40). These rodent studies strongly suggest that disruption of the RPE cell-cell contacts leads to cytokine overexpression, macrophage recruitment and RPE atrophy *in vivo*. Future studies on GzmB-KO mouse models, as well as the apolipoprotein-E KO mouse, a model of accelerated aging (11, 28), may allow us to identify the detailed timeline and mechanism whereby the age-related increase in extracellular GzmB activity leads to cleavage of tight junctional proteins, subsequent RPE atrophy and loss of barrier function *in vivo*.

### Consequences of GzmB Cleavage of ECM and Basement Membranes in BM

RPE cells control the synthesis of all the structural elements in BM (FN, COL I, III-IV, and LAM) (1, 41, 42). Our results showed that RPE-derived FN and LAM-5 were degraded by the application of exogenous GzmB *in vitro*, therefore validating that ocular FN and LAM-5 are substrates of GzmB. GzmB cleavage of COL-IV was present, but did not reach significance. It is possible that COL-IV is partially cleaved by GzmB; however, earlier studies suggest it may be a substrate of GzmA (39).

Preliminary work on human eye-cup preparations incubated in exogenous GzmB demonstrated reduced numbers of adherent RPE, suggesting that ECM degradation can also effect the outer basement membrane potentially leading to RPE anoikis (data not shown) (1, 43). Similarly, extracellular GzmB may cause degradation of the choroidal basement membrane, which supports the endothelium and choriocapillaries, the principal blood supply to the outer retina. The loss of the endothelial cells can cause closure of capillaries and ghost vessels (44–46), which in turn stops the nutrient and gas exchange to the outer retina, altering RPE and photoreceptor function and, eventually, their cell death in GA. Future studies will use an *ex vivo* choroidal explant model to assess the role of exogenous GzmB (and inhibitors of GzmB) on endothelial cell viability, release of angiogenic factors such as VEGF from BM and subsequent choroidal sprouting (47–52).

### GzmB's Putative Role in AMD

Our results demonstrating GzmB expression in both RPE and choroidal mast cells suggests that this serine protease may play multiple roles in the outer retina. Eyes with soft drusen (but without AMD) were more likely to have GzmB+ RPE cells than the other groups studied. These GzmB+ RPE cells appeared to have the RPE phenotype of “non-uniform” described earlier (53). Given that our sample of soft drusen eyes did not display AMD features, the “non-uniform” RPE may represent age-related changes in RPE. We do not yet know if soft drusen upregulates GzmB in nearby RPE cells or whether the degradation of tight junctional contacts between RPE (reported here in *in vitro* studies) may be caused by RPE or mast-cell derived GzmB. Future studies will identify whether soft drusen components upregulate GzmB in RPE cells *in vitro*, and mouse models will help us to clarify further the relationship between extracellular GzmB and the cleavage of ECM and tight junctions in outer retina.

Our results also show that CNV eyes had significantly more GzmB+ cells in the choroid than any other groups tested. Our group showed that GzmB cleaves FN and released VEGF from endothelial cell derived ECM *in vitro* and increased vascular permeability in skin *in vivo* using a Miles/Evan's Blue assay (54). These findings suggest that extracellular GzmB potentially promotes abnormal CNV by: (1) releasing sequestered VEGF from BM; and (2) promoting vascular leakage by disrupting choroidal endothelial cell function. However, future work is needed to understand GzmB's overall role in angiogenesis, as we also showed that GzmB cleavage of FN may also dysregulate angiogenesis by impairing endothelial cell adhesion, migration, and capillary formation resulting in vascular leakage *in vitro* (55).

VEGF plays a key role in CNV and is the target of FDA-approved anti-angiogenic drugs (e.g., Lucentis, Avastin, Eylea). While these drugs are effective, recent studies show a decline in long-term efficacy, which is believed to result from the emergence of VEGF-independent mechanisms involved in exacerbating the abnormal angiogenic milieu in the AMD eye. Moreover, some patients on anti-VEGF drugs do not benefit (e.g., non-responders) and their vision continues to diminish. Insights into a mechanism underlying resistance observed in some AMD non-responders come from a recent article by Wroblewski et al. that showed increased levels of GzmB from mast cells upon anti-VEGF treatment for cancer (tumor angiogenesis) (56). In Wroblewski et al., activity of extracellular GzmB was shown to liberate sequestered pro-angiogenic factors (from the tumor ECM) outside the VEGF-VEGFR2 axis. Increased GzmB also supported endothelial cell migration and vessel formation, and promoted tumor angiogenesis, despite anti-VEGF treatments. Inhibitors of mast cell degranulation increased the efficacy of anti-VEGF therapy in this model, providing supportive evidence that pharmacological inhibition of extracellular GzmB may ameliorate neovascularization in CNV.

Treating AMD is a multidimensional health care problem, with a global cost of vision impairment estimated to be nearly $343 billion worldwide. There is a need for early and prophylactic therapies for AMD. Here we present a novel concept to treat the earliest events in AMD, by targeting extracellular GzmB in outer retina, in order to suppress remodeling of BM and deterioration of the oBRB, two of the earliest events associated with AMD pathogenesis.

## Data Availability Statement

The datasets generated for this study are available on request to the corresponding author.

## Ethics Statement

This study used primary human fetal RPE cells isolated from fetal donor eyes and adult post-mortem human eye tissues consented for research. The studies involving human participants were reviewed and approved by the Clinical Ethics Research Board of the University of British Columbia and strictly adhered to the Declaration of Helsinki. The patients/participants provided their written informed consent to participate in this study. This study used C57Bl6/J mice retina. The animal study was reviewed and approved by UBC Animal Care Committee.

## Author Contributions

JM and YT wrote the manuscript. JM and DG conceived and designed the study, obtained funding, analyzed and interpreted the data, and critically revised the manuscript. YT and JC performed the experiments, analyzed and interpreted the data and generated figures. MZ, SH, and CT provided expert opinion and technical advice on GzmB assays and assisted with study design and interpretation. MZ generated and purified endogenous GzmB from YT cells for use in this study. All authors read and approved the final manuscript.

### Conflict of Interest

DG is a co-Founder and serves as the Chief Scientific Officer of viDA Therapeutics. However, no viDA products were used in this manuscript. The remaining authors declare that the research was conducted in the absence of any commercial or financial relationships that could be construed as a potential conflict of interest.
