# Supplementary figures and images for: Retinal Distribution and Extracellular Activity of Granzyme B: A Serine Protease That Degrades Retinal Pigment Epithelial Tight Junctions and Extracellular Matrix Proteins
Source: Front Immunol. 2020 Apr 7;11:574. doi: 10.3389/fimmu.2020.00574 (PMC7155911; doi:10.3389/fimmu.2020.00574)

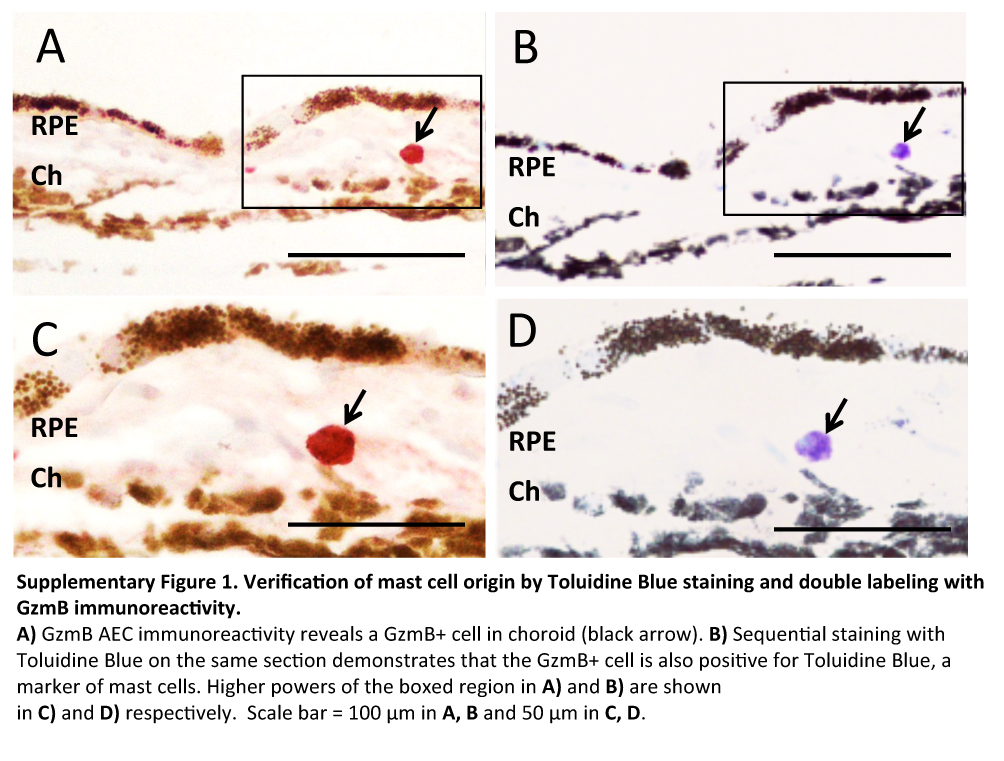

Supplement: Supplementary file 1 [file Image_1.TIF]

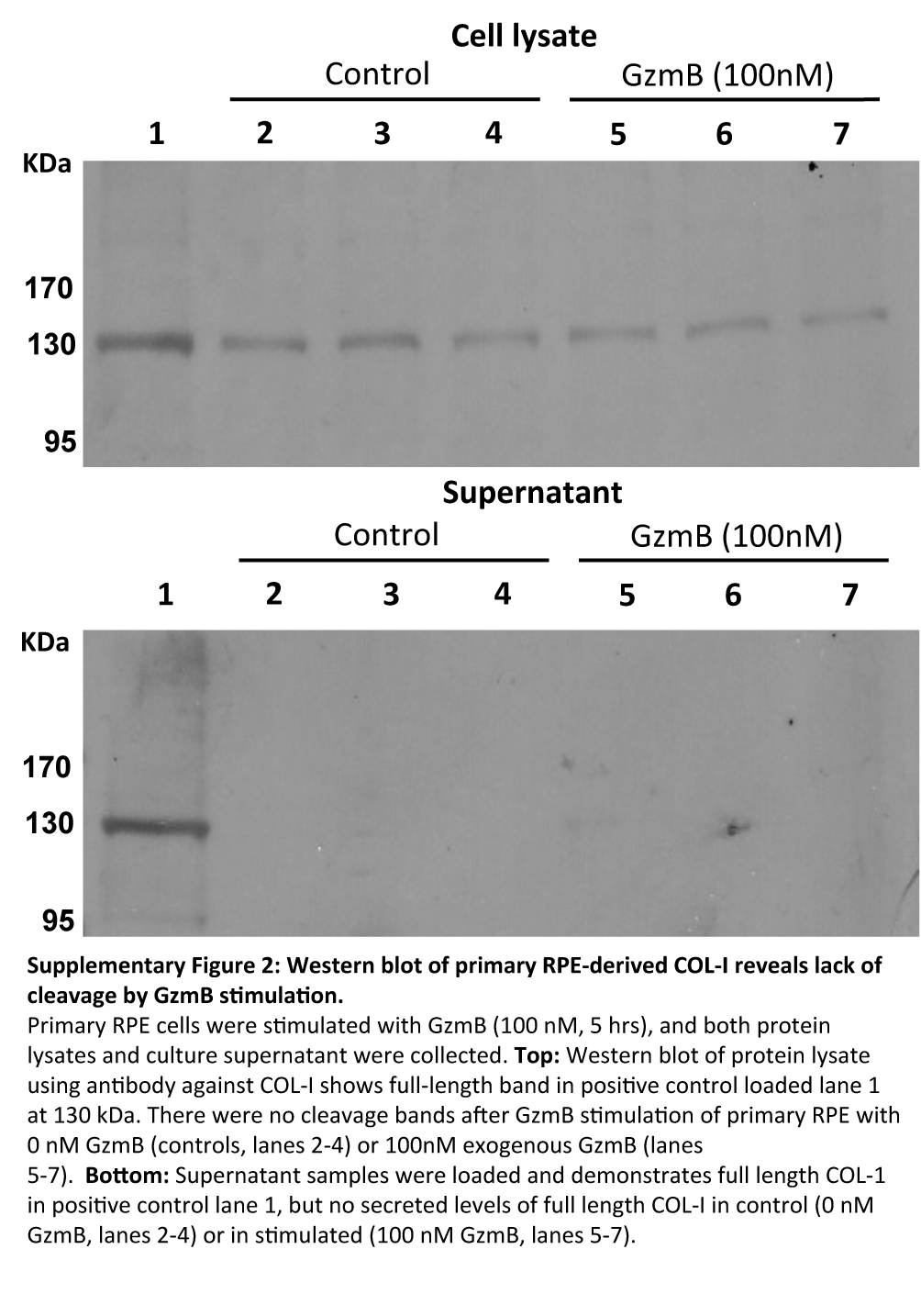

Supplement: Supplementary file 2 [file Image_2.TIF]

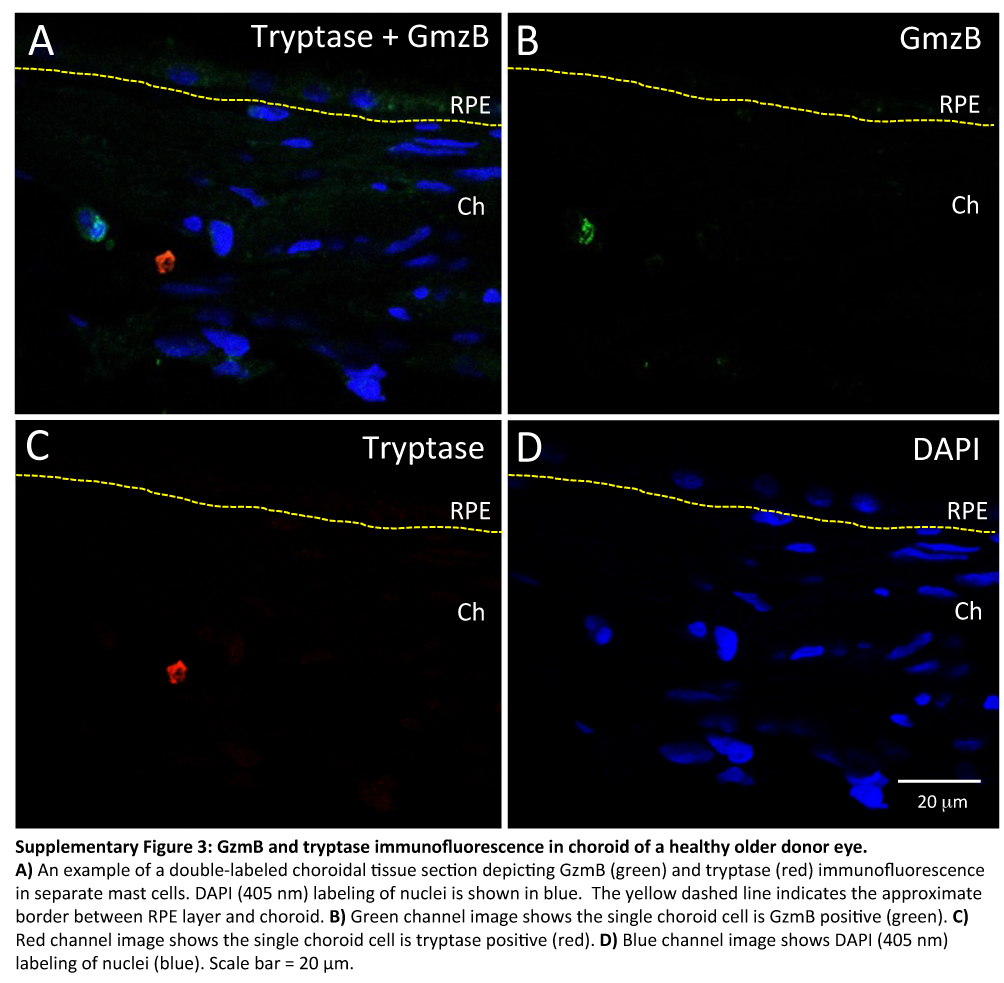

Supplement: Supplementary file 3 [file Image_3.TIF]

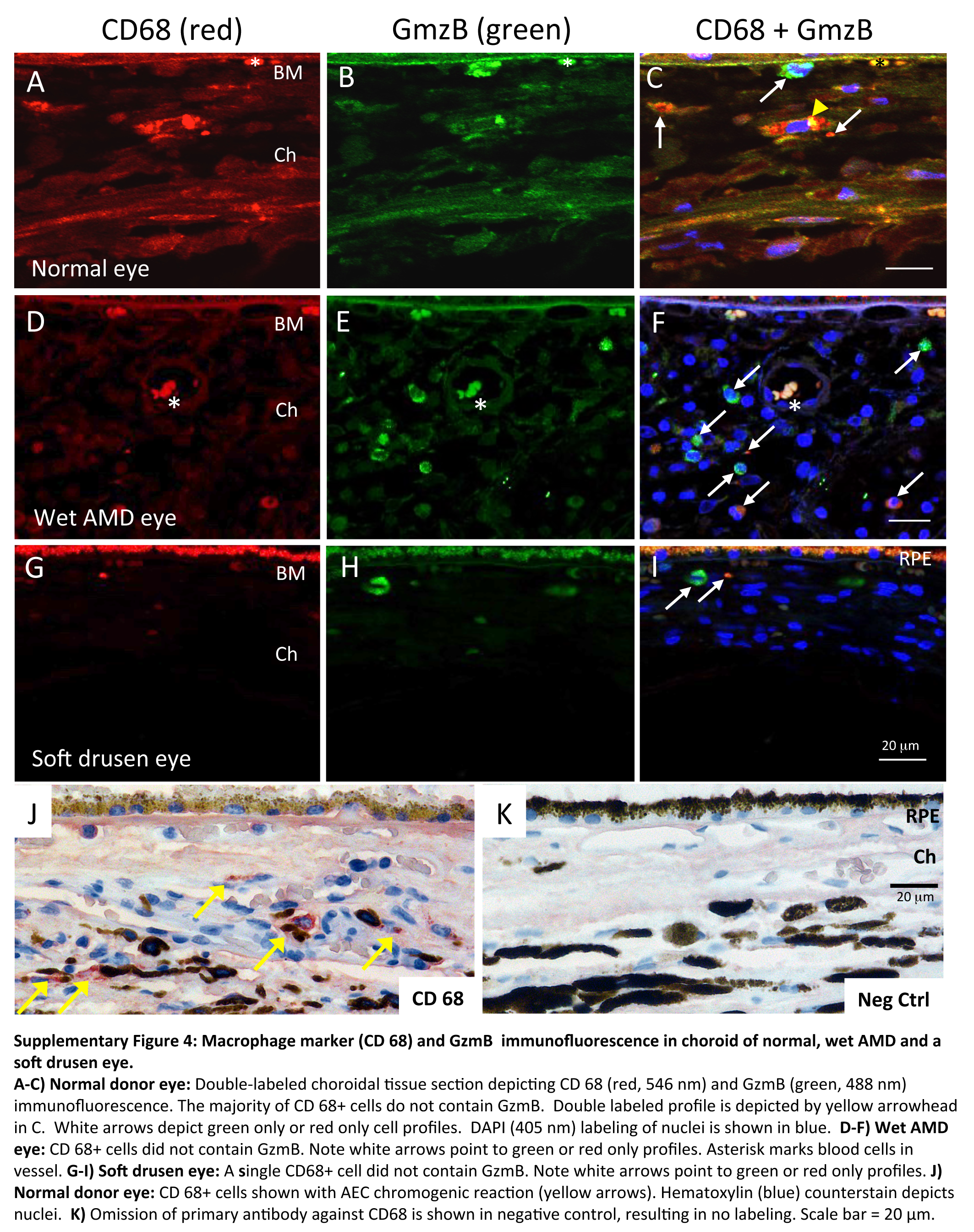

Supplement: Supplementary file 4 [file Image_4.TIF]

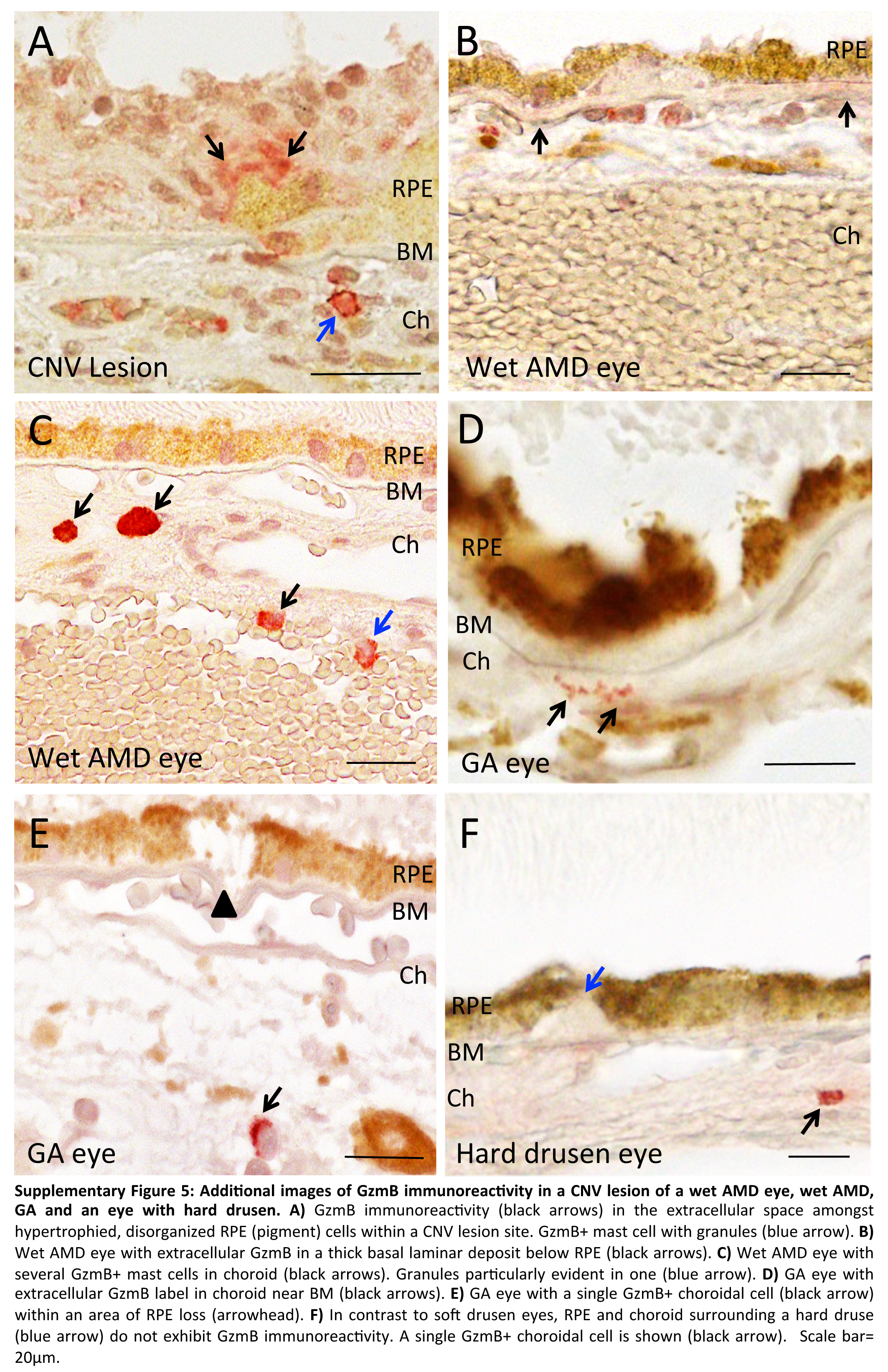

Supplement: Supplementary file 5 [file Image_5.TIF]

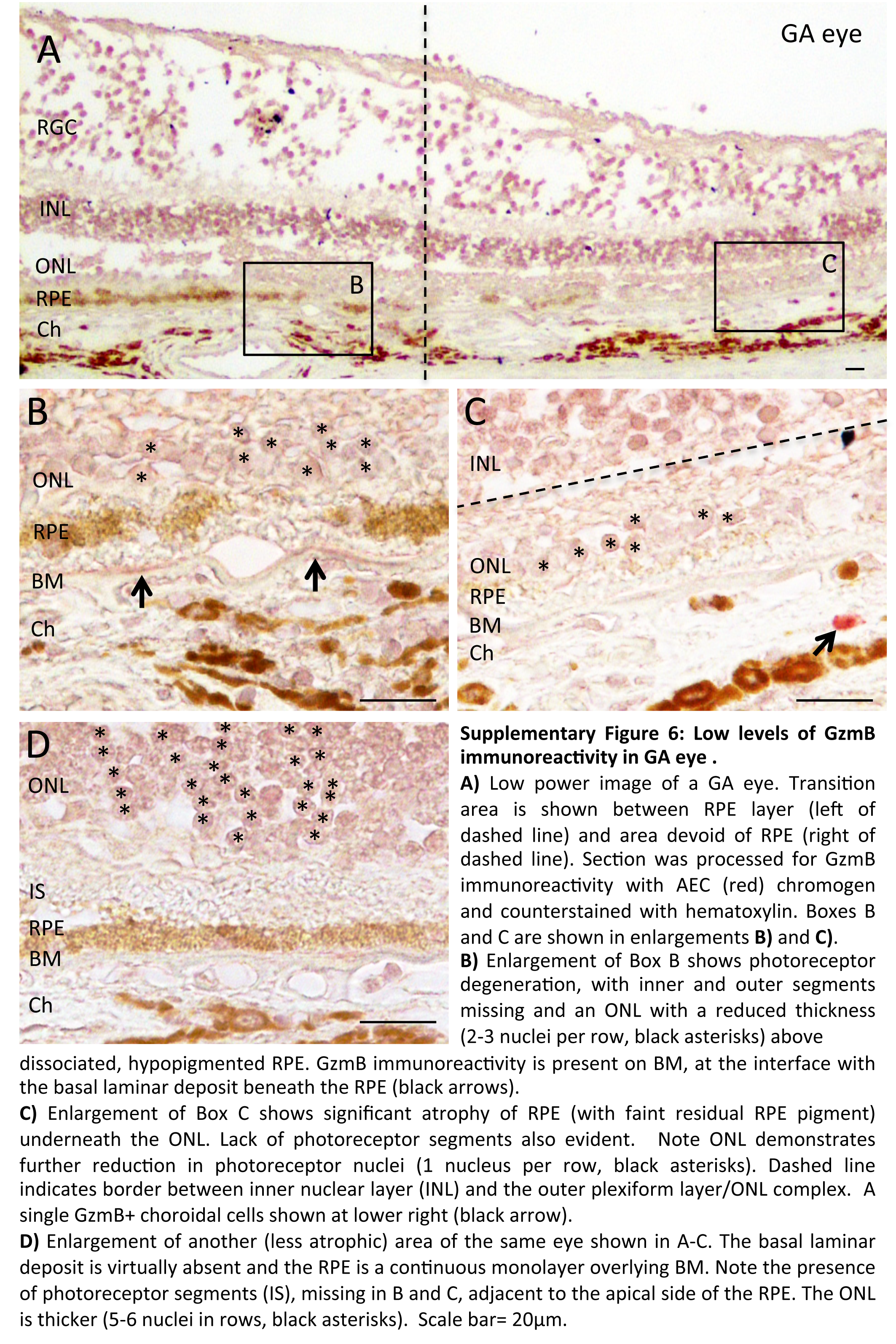

Supplement: Supplementary file 6 [file Image_6.TIF]
